# Watch out! High vigilance at small waterholes when alone in open trees

**DOI:** 10.1371/journal.pone.0304257

**Published:** 2024-07-03

**Authors:** Gerhard Hofmann, Claudia Mettke-Hofmann

**Affiliations:** 1 Moreton, Wirral, United Kingdom; 2 School of Biological & Environmental Sciences, Liverpool John Moores University, Liverpool, United Kingdom; UFERSA: Universidade Federal Rural do Semi-Arido, BRAZIL

## Abstract

An animal’s environment contains many risks causing animals to scan their environment for potential predators and threats from conspecifics. How much time they invest in such vigilance depends on environmental and social factors. Most vigilance studies have been conducted in a foraging context with little known about vigilance in other contexts. Here we investigated vigilance of Gouldian finches at waterholes considering environmental and social factors. Gouldian finches are colour polymorphic with two main head colours in both sexes co-occurring in the same population, black-headed and red-headed. Data collection was done on birds sitting in trees surrounding waterholes by measuring the frequency of head movements, which reflects how frequently they change their field of view, i.e., scan different areas in their environment. A higher frequency generally reflects higher vigilance. Gouldian finches had a higher frequency of head movements when at small waterholes and when sitting in open, leafless trees. Moreover, head movements were higher when birds were alone in the tree as compared to groups of birds. Finally, birds in same head colour morph groups had a higher frequency of head movements than birds in mixed head colour groups. Results indicate heightened vigilance with increased perception of predation risk (small waterholes, open exposed perch, when alone) but that social vigilance also played a role (group composition) with particularly the aggressive red-headed birds being more vigilant when together with other red-headed birds. Future research should investigate the effect of smaller waterholes as global warming will cause smaller waterholes to become more common for longer periods of time, which can increase stress in the birds.

## Introduction

Situations can quickly change in an animal’s environment requiring constant attention. Most animals use vigilance to scan their environment for potential predators [1–4] but also monitor group members to stay with the group [5] or spot challenges early on (social vigilance) [6–8]. Higher vigilance is generally reflected in an increased scan frequency or duration [7, 9–11] and is linked to higher predator detection [12]. However, most studies are done in a foraging context with little known about vigilance in other contexts.

Generally, vigilance increases with increased threat perception [1, 7, 10, 11, 13–16] and is modulated by environmental variables such as openness (up- or down regulated [17–20]) and social variables with group size the most prevalent to reduce vigilance in larger groups [21–24] (many eyes hypothesis, dilution hypothesis [21, 25]; but see [8] for an increase in vigilance with group size to monitor other individuals for potential threats–social vigilance).

Waterholes are dangerous places as they are known to predators and prey must come to drink [26]. Heightened vigilance is key to avoid predation [2, 18]. However, environmental conditions are likely to vary between waterholes resulting in potentially different levels of threat. One important factor is the amount of vegetation around waterholes [27]. Vegetation can provide cover reducing predation risk [28, 29]. However, at the same time it can obstruct a clear view resulting in discovering a predator too late [30]. Votto et al. [27] found that the presence of small and medium-sized canopy foragers at waterholes basically dropped to zero when cover was more than 10 m away reflecting the need for cover, whereas the proportion of cover was negatively related to the number of birds present reflecting the importance of visibility. Hall et al. [18] suggested different vigilance strategies capturing the protection–visibility dilemma. Prey that perceives cover as protection should show reduced vigilance in dense vegetation (refuge vigilance hypothesis), whereas prey perceiving cover as obstruction should increase vigilance in those areas (visibility vigilance hypothesis). In their study, kit foxes (*Vulpes macrotis*) were less vigilant at waterholes with less cover supporting the visibility vigilance hypothesis [18]. Furthermore, vigilance is often higher during approach towards a waterhole as compared to when at the waterhole allowing the animals to assess risk before committing to drink and is higher when predators are present [2, 4]. Finally, larger group sizes have been shown to reduce vigilance at waterholes [2, 26] as has group composition containing other species [2].

Little else is known about vigilance at waterholes when it comes to group composition or differential responses of specific individuals. However, studies investigating vigilance in other contexts found that groups with young often have higher vigilance [8, 31], as can be in mixed sex [8] or mixed morph groups [32]. Moreover, vigilance in a foraging context can differ among group members. Contrasting results have been found for age. Some studies found juveniles being less vigilant as they are often less efficient in finding food and trade-off foraging against vigilance [14, 33, 34], whereas other studies found juveniles to be more vigilant [9]. Conspicuousness also affects vigilance with more conspicuous males being more vigilant [35]. Linked to this, in colour polymorphic species, morphs can differ in their vigilance [36, 37]. Colour polymorphism might hamper detection by predators as detection of uniform prey is easier [38].

Most studies measure the frequency and duration of heads-up [2, 39–41] or inter-scan-intervals between foraging bouts [36] as foraging and vigilance are often in conflict with each other [40]. Another approach is to measure the frequency of horizontal head movements (rather than heads-up) as they provide important information about how often an animal changes its visual field, i.e., looks in a different direction [42]. A high frequency of head movements (and correspondingly short duration of looking in a particular direction) has been linked to higher vigilance as a larger area is scanned in a shorter period of time allowing the detection of any threats (visual search strategy [43]), whereas a lower frequency of head movements represents more a tracking strategy allowing the tracking of targets and assessment of their distance, movement and identification [43]. This method can also be used in other contexts than foraging as it does not rely on the alternation of foraging (heads-down) and vigilance (heads-up; [42]).

The aim of the current study was to investigate vigilance at waterholes in the colour polymorphic Gouldian finch (*Chloebia gouldiae*) considering environmental and social factors. Gouldian finches are endemic to the tropical savannah grassland in North Australia [44]. They are specialised seed eaters, mainly feeding on Sorghum seeds extracted from the ear or picked up from the ground and are nomadic during the non-breeding season to find food [44, 45]. Gouldian finches are listed as endangered by the Australian Department of Environment, Parks and Water Security [46] linked to reduced food availability because of habitat loss and fire management. Adult Gouldian finches are sexually dimorphic with males having a brighter plumage and longer central tail feathers but otherwise looking the same [47]. Juveniles are uniformly gray-green in colour [46]. Gouldian finches occur in three distinct head colour morphs in both sexes in the same population with black-headed birds being most prominent (70%), followed by red-headed birds (30%) and rare yellow-headed birds (< 1%; [47]). Head colour signals the bird’s personality. Black-headed birds are less aggressive than red-headed birds and explore changes in their familiar environment quickly [48, 49], whereas red-headed birds are more likely to venture into new environments and accept new food faster [50, 51]. Moreover, black-headed females are bolder at waterholes than all other birds and take the lead when descending to drink [52]. In the laboratory, vigilance in Gouldian finches is affected by the perceived risk with fewer head movements the more unfamiliar the environment becomes (they adopt a strategy of motionless that has been observed in the wild, too, [32]). Males overall are less vigilant than females and vigilance decreases with age [53]. Group composition affects vigilance with mixed head colour pairings showing higher vigilance than same head colour pairings [32, 53].

In the current study, vigilance of Gouldian finches perched in trees surrounding waterholes of different sizes was investigated. During the dry season, waterholes become increasingly restricted and investigating vigilance of Gouldian finches at waterholes is important to identify challenges, particularly under the current global warming situation. The following predictions were formulated.

1. Environmental factors:
  - Vigilance is higher at smaller waterholes as the threat of predation increases [1, 7, 11, 15]. Smaller waterholes allow fewer choices to select a safer spot to drink and make predator attacks easier. Higher vigilance before descending is required to make sure that the environment is safe. Additionally, there is more competition at smaller waterholes and birds have to increasingly monitor other birds before deciding when to decent and with whom to drink.
  - Vigilance is higher in open trees as birds are more exposed. Gouldian finches sit for extended periods of time in trees before descending to the waterhole [52]. They are likely to use cover as protection and scan the area from there (refuge vigilance hypothesis; [18]).
2. Social factors:
  - Vigilance decreases with group size as has been observed in many other study systems [21–24].
  - Vigilance is higher in mixed head colour groups as found in the laboratory [32, 53].
  - Individual factors such as age, sex or head colour affect vigilance. Based on laboratory findings females might be more vigilant than males and juveniles more vigilant than adults [53].

## Material and methods

### Study species and locations

The study was conducted during the dry, non-breeding season between July and August 2023 in the Kimberley region, Western Australia, which is a stronghold of the species [46]. During this period the Gouldian finch is nomadic following the availability of seeds [44, 45].

Data collection occurred at seven waterholes stretching from Wyndham in the West (15°29’08.3"S 128°07’14.9"E) to Lake Argyle in the East (16°05’55.4"S 128°42’17.1"E) largely along the Great Northern and Victoria Highway. The only location off the Highway was located in the El Questro resort (16°00’29"S 127°58’50"E). Adjacent waterholes were about 12.5 km (median) with a range of 6.8–47.0 km (quartiles) apart but at least 1.5 km (one location; Table 1). The two locations close together were separated by a ridge and were visited by different individuals as numbers and compositions differed between the two sites. It is, therefore, assumed that waterholes are independent sampling units. The habitat across the entire study area and around all waterholes was similar characterised by open eucalyptus woodland comprising of a mixture of eucalyptus (*Eucalyptus spp*.), bloodwood (*Corymbia spp*.) and boab (*Adansonia gregorii*) trees with annual sorghum grass (*Sorghum spp*.) on the ground typical for the species [54]. Waterholes largely originated from dried-out creeks but could also be from man-made structures and differed in size ranging from < 1 m^2^ to stretches of creeks of 100 m lengths and 10 m width (Table 1). All waterholes had open trees without leaves (boabs or dead trees) and trees with foliage providing cover. Predator attacks by two of the main avian predators, the Brown falcon (*Falco berigora*) and the Brown goshawk (*Accipiter fasciatus*), were observed daily at all waterholes. Waterholes were selected based on long-term use (pers. comm. Gary Fitt), local knowledge and finding new waterholes used by Gouldian finches.

**Table 1:** Distance between waterholes and waterhole size to investigate vigilance in Gouldian finches in the Kimberley region, Northern Australia.

Location	Distance between adjacent sites (km)	Waterhole size (m)	Number of observations	Number of focal birds
**1**	11.1	0.5 x 1	6	207
2*	13.8	32 x 104	2	9
**3**	1.5	0.5 x 1	5	56
**4**	8.6	3 x 6	5	35
**5**	38.8	12 x 39	4	17
**6**	80.6	6 x 35	7	63
7*		0.5 x 1	4	32

The value presented in the column distance between adjacent sites is the distance to the site next in the table (11.1 km from site 1 to site 2); * indicates man-made waterholes.

### Data collection

Each location was observed two to seven times depending on number of birds visiting the waterhole. Visits were on average 3.8 days ± 1.9 days apart. Data were collected by the same observer (CMH) throughout the study period between 5:30 am and 10:00 am with some additional data points outside this window. Gouldian finches visit waterholes once a day, usually in the morning [55]. Therefore, we assume that we sampled different birds on a given day. The observer was located about 10–15 m away from the water depending on available cover observing the birds with a binocular and recording all data on a Dictaphone (Sony IC Recorder ICD-PX440). Data collection started as soon as a Gouldian finch landed in a tree. Head colour, sex and age of the focal bird were recorded alongside the number and identity (head colour, sex, age) of other Gouldian finches in the tree. The number of other Gouldian finches was restricted to the same or neighbouring tree of the focal bird as we assumed that dilution effects [21] would work primarily when birds were in relatively close proximity to each other. In most cases this coincided with the overall number of birds present. We made a note when Gouldian finches were sitting in trees further away but did not consider this further as it only happened with large groups. The openness of the tree the bird was in was also recorded ranging from open to medium to dense (see below). With the Dictaphone running each head movement was counted talking quietly into the Dictaphone. A head movement was defined as any head movement (horizontal or vertical) as each movement brings the bird’s eye in contact with a different part of the environment [42]. When Gouldian finches land in a tree, they remain sitting in the same location for extended periods of time (often several minutes) before they fly down to drink. This allowed counting head movements in the same position. When 20 head movements were reached counting started again for logistic reasons. Ideally, three blocks of 20 head movements were recorded. This approximates to about one minute of vigilance data corresponding to other studies (8–310 s [26], at least 15 s [30], 2 min [4], average length 68 s [56]). In case, the focal bird changed position and moved into a different category of cover counting started again. Likewise, counting was stopped when the bird started to preen its plumage and commenced again after preening had finished. When three blocks of vigilance had been recorded, the area was scanned to assess the number and identity of Gouldian finches again. After this, a different focal bird was selected, and the procedure repeated until vigilance of all Gouldian finches present in the tree had been recorded or the birds flew away. To avoid resampling the same bird within a recording session, two different strategies were followed depending on how many individuals of a given head colour, sex and age had been recorded already at that waterhole (during past observation days or observations conducted earlier the day). Either a bird of a different head colour, sex or age was chosen or as many of the same head colour and sex were chosen consecutively to get data of as many different individuals of a particular category as possible. This was possible as birds usually stayed in one position.

### Data analysis

All analyses were done with IBM SPSS Statistics v 29.0.1.0 and the data are available in S1 Table. We extracted the time it took to make twenty head movements or fewer in case the bird changed cover or flew away from the Dictaphone recording. To calculate the frequency of head movements for each individual, the three blocks of uninterrupted head movement recordings were summed up as was the corresponding time it took to make these head movements. The sum of head movements was then divided by the sum of time to get head movements per time. Recordings of at least 10 head movements were included for further analyses resulting in an overall sample size of 419 birds with a mean of 38.7 ± 0.9 (SE) head movements per individual lasting on average 55.5 ± 1.6 seconds. Head movements per time were LG10 transformed to get normally distributed data.

We used General Linear models (GLM) to investigate vigilance in relation to two environmental and three social factors with frequency of head movements as the dependent variable using an identity link function. The two independent environmental factors were waterhole size (3 levels: small (< 1 m^2^, n = 295), medium (1 m^2^–5 m^2^, n = 35), large (> 10 m^2^, n = 89)) and openness (2 levels: open (trees without leaves, usually boabs, n = 215), cover (combining medium and dense covers, trees with leaves that provided cover, n = 204)). The three independent social factors were morph (3 levels: black-headed (n = 219), red-headed (n = 76), grey (juvenile, n = 124)), sex (3 levels: males (n = 168), females (n = 127), unknown (juvenile, n = 124)) and number of birds present at the start of the vigilance recording (5 levels: 1 = alone (n = 199), 2 = 2 birds (n = 102), 3 = 3–4 birds (n = 72), 4 = 5–7 birds (n = 27), 5 = > 7 birds (n = 19)). For group size categories, it was tried to retain as much information by also considering sample sizes. While categories four and five have relatively small sample sizes, they were kept separate as groups of up to seven birds are not unusual for family groups and might be perceived differently than larger groups of unknown birds Age was not included as a separate variable as juveniles were represented in the morph and sex variable. Finally, the interaction morph x sex was included. Bootstrapping (10,000 samples) was used stratified by location to account for potential resampling of birds across days at the same waterhole and the same bird when observed in different openness levels. Bootstrapping does not require strong distributional assumptions and calculates distributional values using resampling with replacement [57]. The model was validated with the Omnibus test comparing the fitted model against the intercept only model.

A second GLM was run to test for effects of group composition on vigilance in adult birds considering only groups of two or more birds (n = 177). Frequency of head movements was the dependent variable with an identity link function. Two independent variables were included: Group composition (2 levels: same head colour (n = 109), mixed head colour (n = 68)) and morph (2 levels: black-headed (n = 131), red-headed (n = 46)). The interaction term group composition x morph was also included as laboratory studies found differences in vigilance depending on morph [32]. The same bootstrapping procedure was used as before.

### Ethics

All applicable international, national, and/or institutional guidelines for the care and use of animals were followed. The study was conducted under a Notification of Observation permit (F18979-05) by the University of Western Australia, Perth, WA, Australia. Permissions to collect data were given by the Shire of Wyndham East Kimberley Council Wyndham branch, El Questro Resort and Lake Argyle Homestead.

### Inclusivity in global research

Additional information regarding the ethical, cultural, and scientific considerations specific to inclusivity in global research is included in the S1 Checklist.

## Results

**Fig 1:**
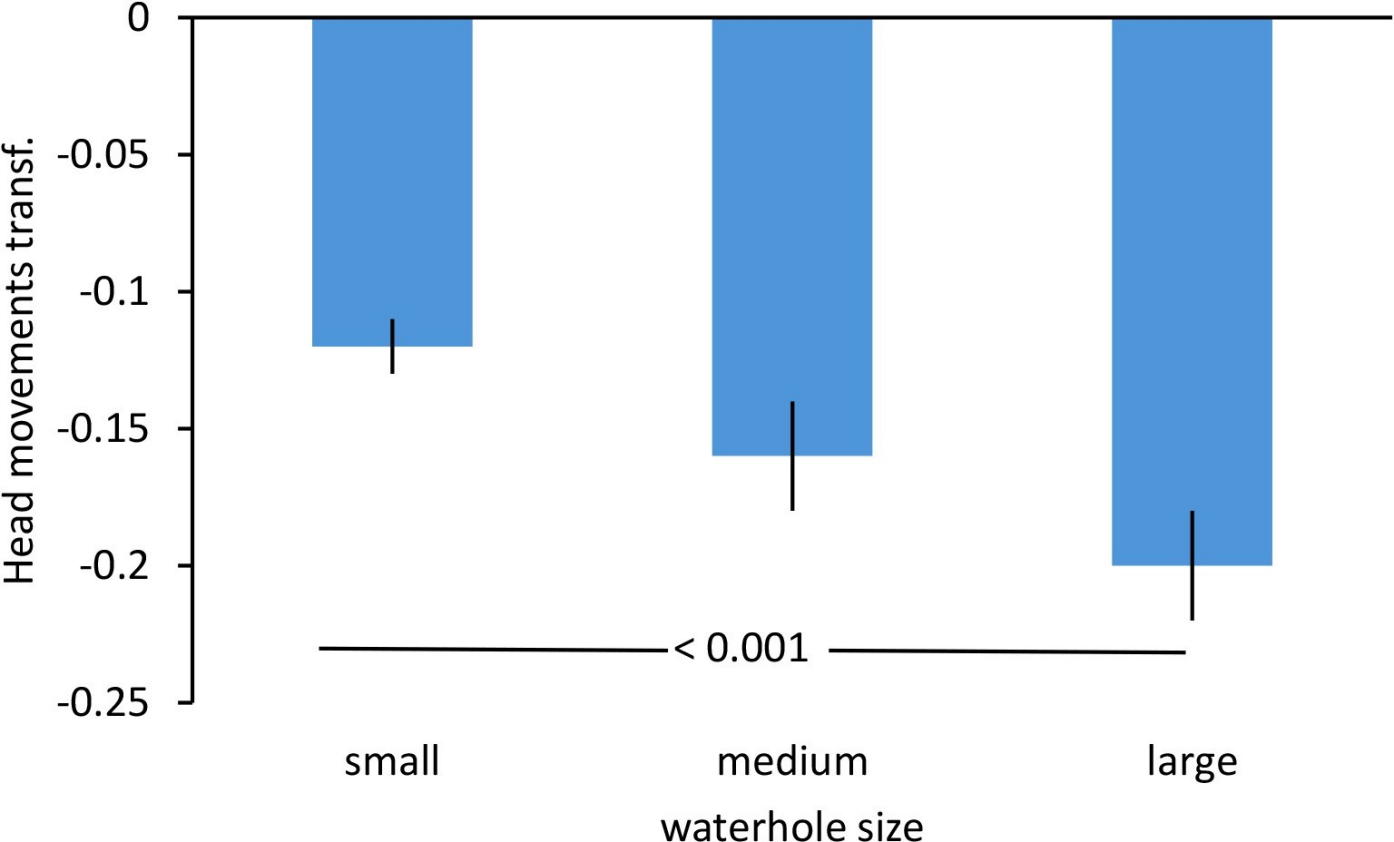
Vigilance of Gouldian finches in relation to waterhole size in the Kimberley region, Northern Australia. Mean ± SE of frequency of head movements in trees is shown in relation to different waterhole sizes (small: < 1 m^2^, medium: 1–5 m^2^, large: > 10 m^2^). Smaller values represent a lower frequency of head movements. Numbers in the figure represent p-values. Transf.: transformed data.

The General Linear Model testing vigilance of Gouldian finches in trees around waterholes in relation to environmental and social factors showed that the fitted model was significantly better than the intercept only model (Omnibus test: χ^2^ = 60.237, df = 11, p < 0.001). Both ecological variables were significant (Table 2). The size of the waterhole significantly affected vigilance (Table 2) with a higher frequency of head movements the smaller the waterhole (Fig 1). Parameter estimates with bootstrapping showed that the frequency of head movements at small waterholes was significantly higher than at large waterholes (Bootstrap: p < 0.001), whereas no significant difference was found between small and medium ones (p = 0.137).

**Table 2:** Outcome of the GLM with bootstrapping to investigate the effect of ecological and social factors on the frequency of head movements in Gouldian finches at waterholes in the Kimberley region, Northern Australia.

Variable	Wald chi^2^	Df	p-value
**Intercept**	175.902	1	< 0.001
**Waterhole size**	21.242	2	< 0.001
**Openness**	6.674	1	0.010
**Group size**	10.630	4	0.031
**Head colour morph**	0.110	2	0.740
**Sex**	0.363	2	0.547
**Head colour morph x sex**	3.146	1	0.076

Df: degrees of freedom

**Fig 2:**
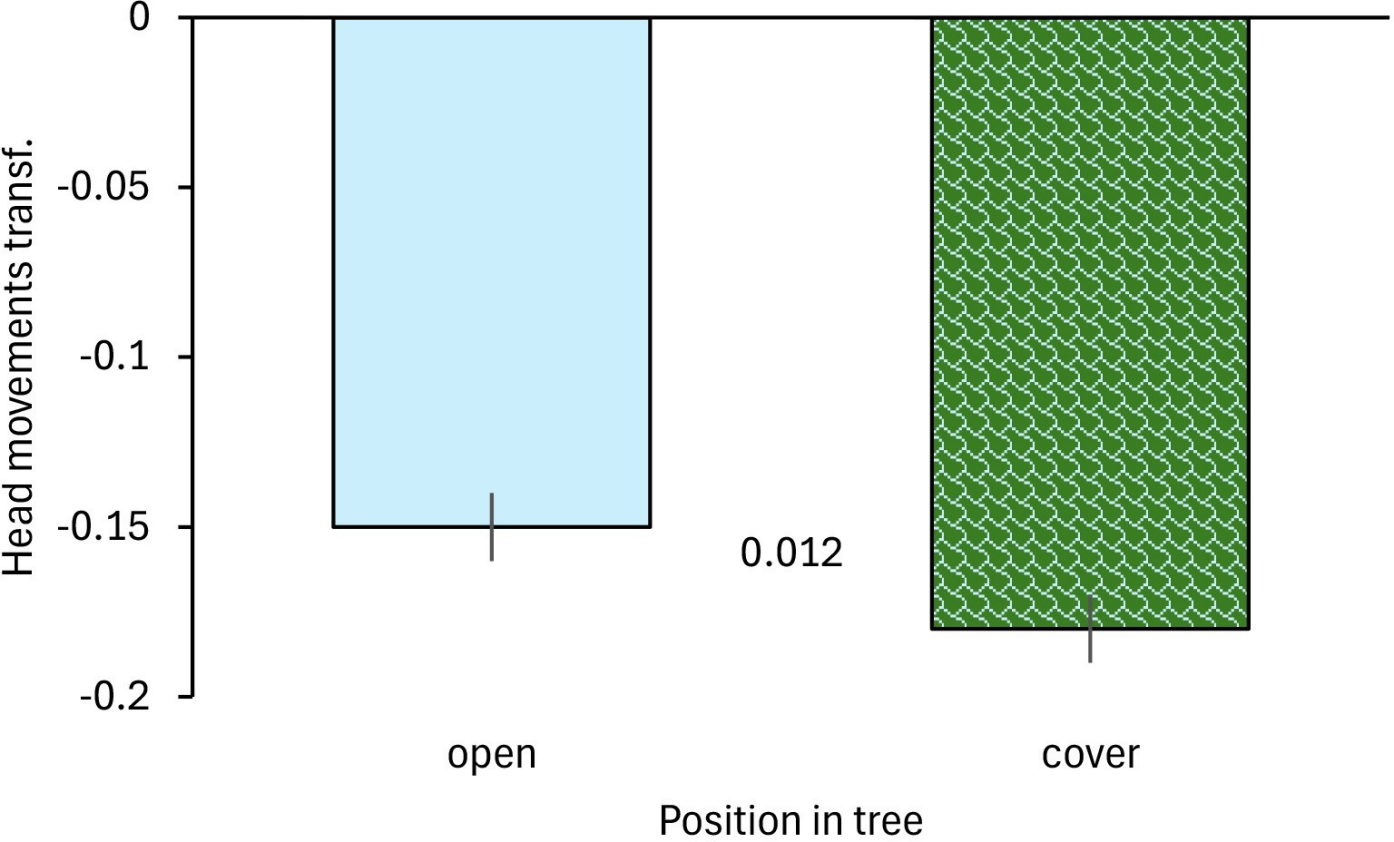
Vigilance of Gouldian finches in relation to exposure level of the birds at waterholes in the Kimberley region, Northern Australia. Mean ± SE of frequency of head movements in trees is shown in relation to openness of the tree (open: tree without leaves, cover: tree with leaves). Smaller values represent a lower frequency of head movements. Numbers in the figure represent p-values. Transf.: transformed data.

Furthermore, openness of the tree the birds were sitting in affected vigilance (Table 2) with a higher frequency of head movements in open (leafless) trees (bootstrapping: p = 0.012; Fig 2).

**Fig 3:**
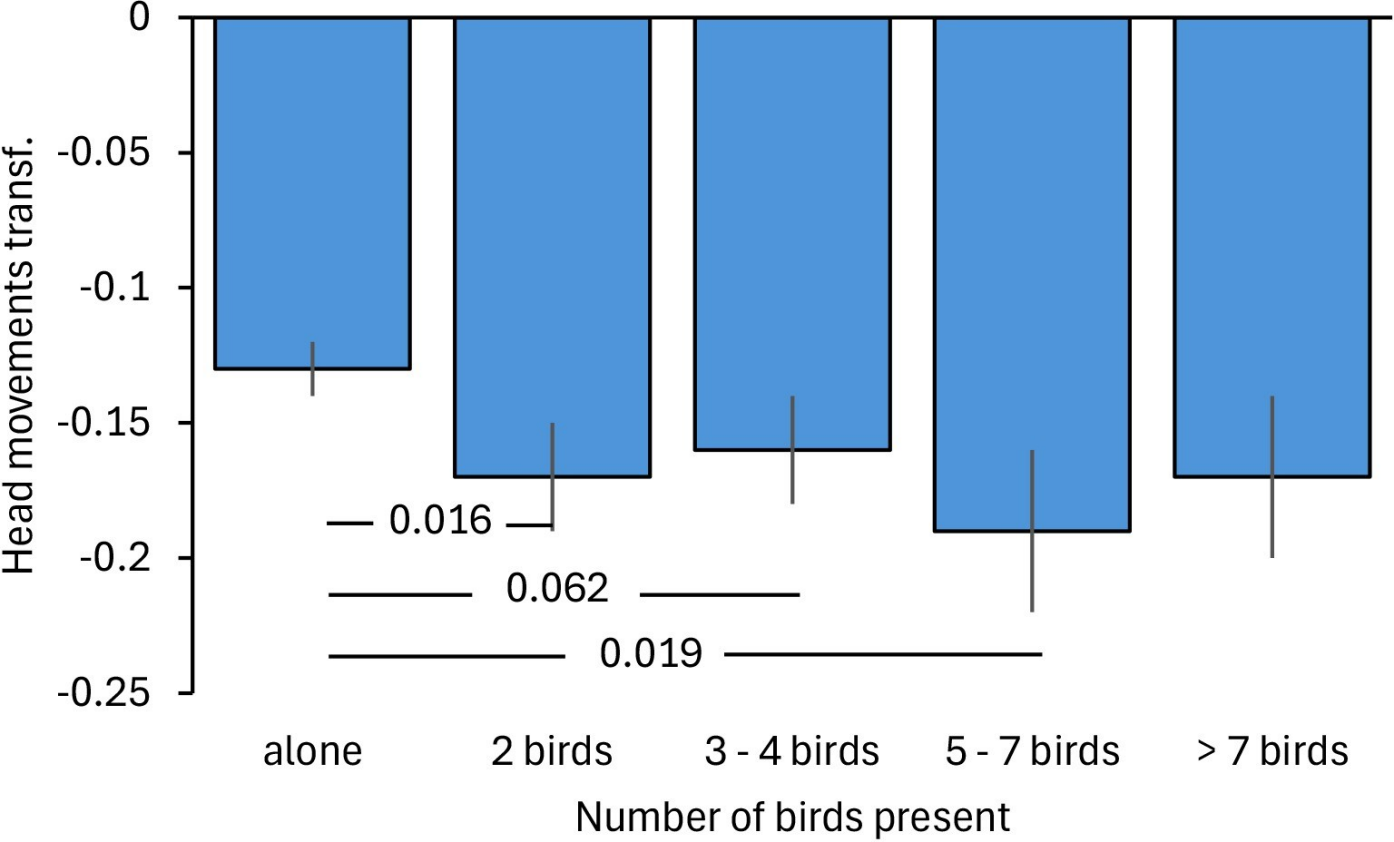
Vigilance of Gouldian finches in relation to group size at waterholes in the Kimberley region, Northern Australia. Mean ± SE of frequency of head movements in trees is shown in relation to group size. Smaller values represent a lower frequency of head movements. Numbers in the figure represent p-values. Transf.: transformed data.

Of the three social factors, only group size had a significant effect on vigilance (Table 2). Single Gouldian finches had a higher frequency of head movements than most other group sizes with significant differences to group sizes of two and 5–7 birds and approaching significance for group sizes of 3–4 birds (bootstrapping: 1 vs 2 birds: p = 0.010, 1 vs 3–4 birds: p = 0.062, 1 vs 5–7 birds: p = 0.019, 1 vs > 7 birds: p = 0.169; Fig 3).

**Fig 4:**
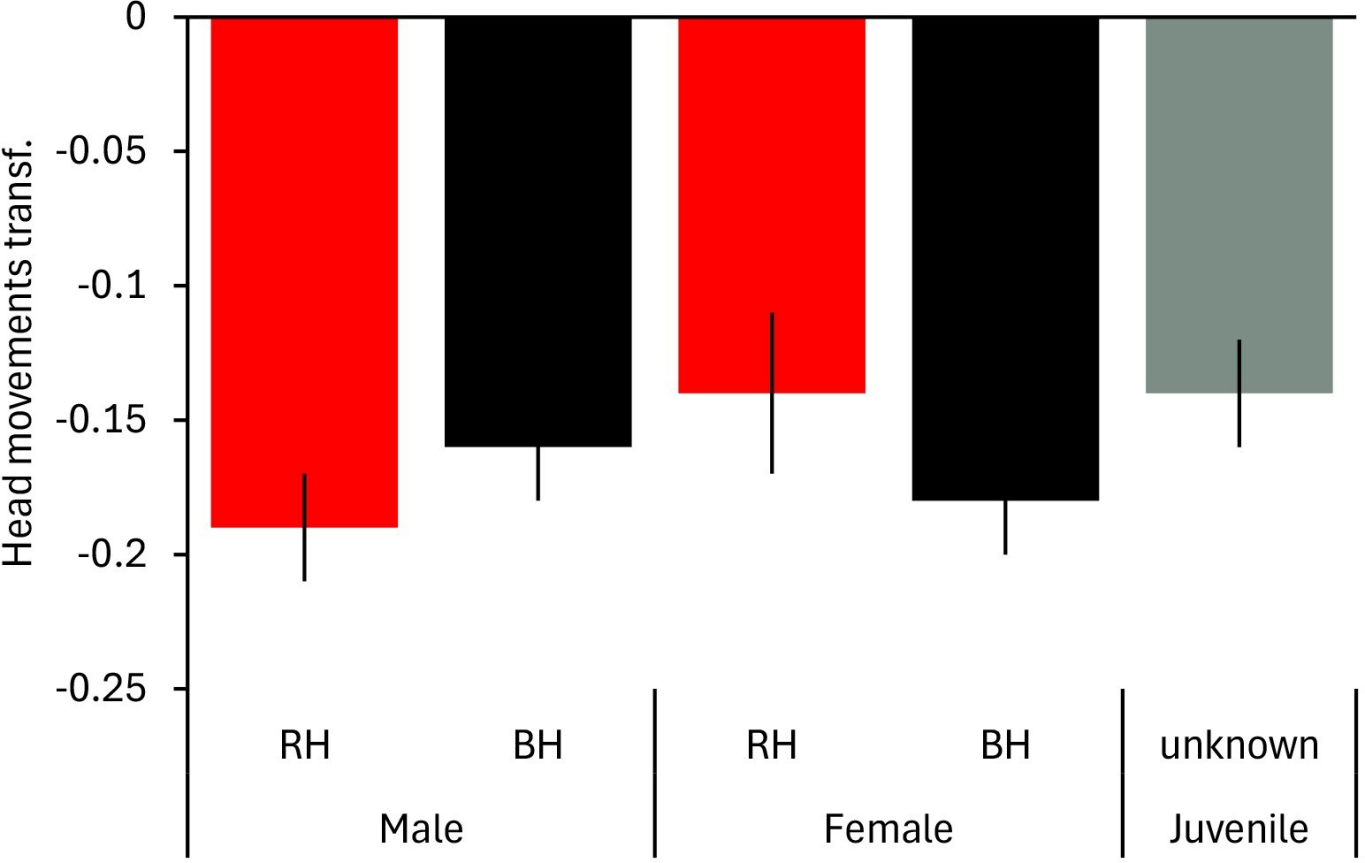
Vigilance of Gouldian finches at waterholes in the Kimberley region, Northern Australia, in relation to head colour morph and sex. Mean ± SE of frequency of head movements in trees is shown in relation to head colour morph (BH = black-headed bird, RH = red-headed bird) and sex. Smaller values represent a lower frequency of head movements. Transf.: transformed data.

The interaction between head colour morph and sex approached significance (Table 2) with black-headed males showing a lower frequency of head movements than juveniles (bootstrapping: p = 0.030; Fig 4).

**Fig 5:**
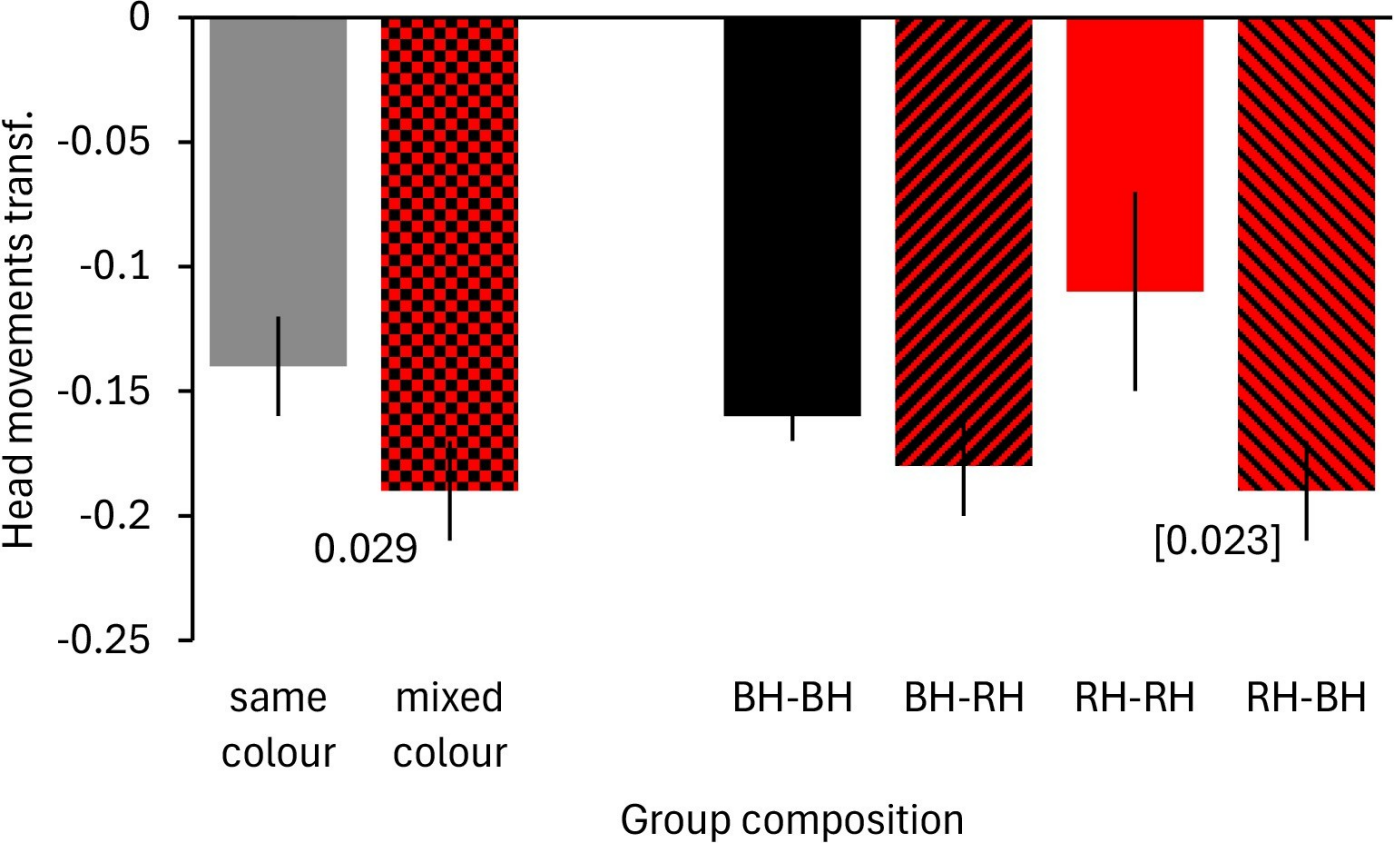
Vigilance of Gouldian finches at waterholes in the Kimberley region, Northern Australia, in relation to group composition. Mean ± SE of frequency of head movements in trees is shown in relation to group composition (same head colour morph vs mixed head colour morph groups) and head colour morph. The left part shows the overall difference between groups of different morph composition, whereas the right part shows this broken down by head colour (BH = black-headed birds, RH = red-headed bird). Smaller values represent a lower frequency of head movements. Numbers in the figure represent p-values. The number in brackets indicates that the interaction term linked to this posthoc result was not significant. Transf.: transformed data; BH-BH: focal black-headed bird in groups of other black-headed birds, BH-RH: focal black-headed bird in groups of red-headed birds, RH-RH: focal red-headed bird in groups of other red-headed birds, RH-BH: focal red-headed bird in groups of black-headed birds.

The second GLM to investigate the effect of group composition in adult Gouldian finches and group sizes of two or more birds showed that groups consisting of mixed head colour morphs made fewer head movements than groups of same head colour morphs (Table 3; Fig 5). However, this fitted model was not significantly different from the intercept only model (χ^2^ = 4.800, df = 3, p = 0.187). The interaction group composition x head colour morph was not significant (Table 3) but it should be mentioned that red-headed birds in same head colour morph groups made more head movements than red-headed birds in mixed head colour morph groups (bootstrapping: p = 0.023).

**Table 3:** Outcome of the GLM with bootstrapping to investigate the effect of group composition on the frequency of head movements in Gouldian finches at waterholes in the Kimberley region, Northern Australia.

Variable	Wald chi^2^	Df	p-value
**Intercept**	185.549	1	< 0.001
**Group composition**	4.785	1	0.029
**Head colour morph**	0.844	1	0.358
**Group composition x head colour morph**	1.606	1	0.205

Df: degrees of freedom

## Discussion

Vigilance of Gouldian finches in trees around waterholes was affected by ecological and social factors. Vigilance was higher at small waterholes and when the birds were sitting alone in an exposed position. Moreover, birds in groups of same head colour morph showed higher vigilance than birds in groups of mixed head colour morph.

Gouldian finches were more vigilant at small as compared to large waterholes confirming prediction 1a. While small waterholes might be more difficult to find by a predator, once they are discovered predators can focus their attention on a small area, which likely increases predation success. Smaller waterholes, therefore, represent a higher threat to prey than larger waterholes and prey might be more vigilant and scan the area thoroughly before descending. This corresponds to higher vigilance in ungulates when approaching and leaving waterholes with increased hunting pressure as compared to the period of drinking when vigilance did not differ between areas with/without hunting [4]. In both studies, animals assessed risk before committing to drink allowing decision making about whether or not to drink in the first-place weighting costs and benefits. Higher vigilance at small waterholes indicates that the birds perceived them as more threatening in line with many other studies showing higher vigilance in more threatening situations across species [1, 7, 10, 11, 13–16]. Whether the Gouldian finches were also more likely to leave small waterholes without drinking is unknown. However, this would be an interesting question to follow up. Moreover, higher vigilance before descending to the waterhole might allow synchronisation with others [4], which is more important at small waterholes (dilution effect [21]). Alternatively, birds might scan their environment for other birds (social vigilance; [6, 8, 10, 29]) as small waterholes only provide limited space to drink. Assessing the number and type of competitors might be important to decide when to descent to drink. Atkins et al. [10] found that the number of geese being vigilant increased with increasing group size under increased predation pressure possibly linked to social information gathering, whereas it decreased with group size with lower predation pressure. The link between higher vigilance and smaller waterholes is concerning as with global warming waterholes will dry out more quickly and birds have to gather at small waterholes over a longer period in the dry season [27], which could induce stress due to heightened predation pressure and increased competition between birds. This is an area that should be followed up.

Openness of the tree where the birds were perched affected their vigilance with higher vigilance in open trees without leaves. This supports prediction 1b which posed that Gouldian finches would feel safer with more cover (refuge vigilance hypothesis; [18]). Gouldian finches are highly conspicuous and sitting in exposed positions makes them a strong target for predators [58, 59]. Indeed, predation on more conspicuous individuals has been shown in other species [60–62]. Moreover, background matching is often used as an antipredation strategy and reduces predation [63–66]. Sitting in trees with leaves camouflages the Gouldian finches better, particularly as their back is green. Laboratory studies found that Gouldian finches prefer green backgrounds over white ones due to being more camouflaged towards the former [67]. However, it should be mentioned that Gouldian finches did not avoid open trees but often landed there first and only later moved to more cover. This supports to some extent that visibility is important to assess predation risk at a site [27, 29, 30, 68]. Differences in vigilance when sitting in open trees or under cover might also arise when birds initially land in open trees and later move into cover as vigilance is higher at arrival to assess the current situation and declines once the situation has been assessed [69]. However, this is unlikely to be the case here as birds repeatedly moved between trees of different cover and vigilance changed markedly corresponding with the cover (higher vigilance when in open trees).

Group size was the only social factor affecting vigilance with higher vigilance when alone in the tree in relation to most other group sizes. This supports prediction 2a and is in line with many other studies [21–24, 70, 71]. Larger groups provide protection against predation as more individuals scan the environment (many eyes hypothesis; [25]) and the likelihood to be targeted decreases the more individuals are present (dilution effect hypothesis; [21]). Interestingly, differences in vigilance were restricted to single birds versus two or more birds with no effect between small and larger groups (Fig 3) consistent with findings in several European finch species [72]. Possibly, social vigilance increased with increasing group size annihilating any positive effects of increasing groups linked to predation. As mentioned earlier, birds might monitor others to decide with whom to drink and potentially to synchronise descending to the waterhole with others to reduce predation [4]. An increase in social vigilance with increasing group sizes has been found in other bird and mammal species [73]. Likewise, semipalmated sandpipers (*Calidris pusilla*) increased vigilance when neighbouring sandpipers were close to avoid attacks [74]. In Egyptian geese (*Alopochen aegyptiaca*) vigilance increased with group size under high predation with individuals potentially copying vigilance behaviour of more experienced birds [10]. Finally, mallards (*Anas platyrhynchos*) reduced vigilance with increasing group size of other mallards but not when in mixed species flocks with Greylag geese (*Anser anser*) [75].

Predictions about individual differences (prediction 2c) were partly confirmed. While sexes and head colour morphs did not differ in their vigilance, there was a trend for juveniles being more vigilant than adults. This links to findings in the lab with vigilance decreasing from one-year old individuals to older ones [53]. It is also in line with findings in Florida scrub-jays (*Aphelocoma caerulescens*) where juvenile sentinels’ look duration was shorter than in adult sentinels [76]. Juveniles have less experience and might still have to get familiar with threats around waterholes whether from predators or other birds [76]. This might be particularly the case as data collection was done when most of the juveniles were moving around independently from their parents. A study on risk-taking at waterholes also found that juvenile Gouldian finches hesitate longer than adults to drop down for drinking supporting the idea that they are less experienced and feel unsecure around waterholes [52]. Higher vigilance in juveniles has also been found in marmots (*Marmota flaviventris*) after alarm calls, whereas they did not differ in vigilance from adults in control conditions [9]. While vigilance in juveniles is often reported to be lower [14, 33, 34, 77], a meta-analysis found that juveniles are more responsive to alarm calls than adults [78]. The heightened risk at waterholes might have a similar effect in the Gouldian finches.

Finally, when analysing vigilance in adults and group sizes of two or more birds, we found that birds in same head colour morph groups were more vigilant than birds in mixed head colour morph groups, although this model was not significantly different from an intercept only model. Findings, therefore, require future confirmation. The results are opposite to prediction 2b and findings in the laboratory [32, 53]. Possibly, social vigilance played a much larger role in the laboratory with both, black-headed and red-headed birds watching each other in mixed pairings. In the wild, anti-predator vigilance is likely more important, which might change results. Animals in mixed species assemblages often have lower vigilance as compared to single species assemblages [7, 21, 79, 80], which has been explained with differences in perception abilities resulting in earlier detection of threats [81]. Similarly, morph-specific differences in vigilance might exist, which was originally predicted in Mettke-Hofmann [32]. Mixed morphs would, therefore, benefit from each other like mixed species assemblages as they might scan the environment differently, which allows reducing vigilance overall. Additionally, Karpestam et al. [38] proposed that predator have more difficulties to detect prey with different colour patterns potentially providing more protection to prey when in mixed morph groups. The effect was stronger in red-headed birds than in black-headed birds. Red-headed Gouldian finches are more aggressive than black-headed individuals [49, 82, 83] and social vigilance might have been heightened in pure red-headed groups to avoid aggression, whereas risk of aggression for red-headed birds in mixed morph groups would be reduced.

In conclusion, vigilance increased with perceived predation risk in relation to environmental (waterhole size and openness of position) and social factors (group size). The increased vigilance at small waterholes requires further investigation as increases in frequency and duration of heat waves will cause water bodies to shrink faster forcing animals to drink at small waterholes earlier in the season and over a longer period, which could cause stress. Gouldian finches also showed social vigilance as indicated in the effect of group composition with red-headed birds being more vigilant when in same head colour morph groups.

## Supporting information

S1 Checklist Inclusivity in global research questionnaire. (DOCX)

S1 Table Data file. (XLSX)
